# Membranous Croup, Treated by Cauterization of the Fauces and Larynx with Nitrate of Silver

**Published:** 1849-03

**Authors:** 


*Membranous Croup, treated by Cauterization of the Fauces and Larynx with Nitrate of Silver.* By Dr. CLARK.

Dr. Clark had treated five cases in this way: three had recovered—two had died.

*Aug.* 14. *Case.* I.—A boy two and a half years of age, who had been ill two or three days, and had had croupy respiration for thirty-six hours before he was seen. Cauterization was practiced with some alleviation of suffering, but death ensued on the third day.

*Case* II.—A girl five years of age and of scrofulous diathesis. The croupy respiration was well marked, and had been present more than twenty-four hours. Cauterization was employed, and much false membrane was expectorated at various intervals. Considerable relief was obtained in this way; but the child died on the fourth day, with symptoms of exhaustion rather than of suffocation.

*Case* III.—A delicate girl of three and a half years, who had sore throat with hoarseness. False membranes existed in patches on the tonsils, &c.; cauterization was employed. Croupy respiration, however, came on the next evening, but it was relieved in twelve hours, by the expectoration of small fragments of false membrane streaked with blood. The paroxysms of dyspnœa returned several times, being terminated in the same way, and the patient ultimately recovered, although the voice was not wholly restored for three weeks.

*Case* IV.—A child of four years. The symptoms in this case were not very severe, although quite distinct. The croupy breathing had continued without remission for twelve hours—false membranes were remarked in the throat. The treatment here was followed by gradual and speedy recovery, without the expulsion of any large amount of false membrane.

*Case.* V.—The patient, a healthy girl of six years, was seen early in the disease, which lasted a fortnight. She had at first a sore throat and a febrile attack, which disappeared the second day. The only symptom which persisted was a slight and increasing hoarseness, and patches of lymph were to be seen on the tonsils and spreading thence back toward the larynx. These increased until the whole throat was invested with a coating of the lymph and the croupy respiration had supervened. The caustic was regularly applied from the beginning, and the expectoration of the membrane began almost as soon as the breathing became much embarrassed. The paroxysms of distress and the intervals of relief followed each other in this way for nearly three days, when the respiration slowly assumed its natural character. The voice could not be raised above a whisper for several days later.

No medicine but Dover's powder was given, and this was repeated often enough to keep the patient constantly under its influence. The caustic was of the strength of xl grs. to ℥j of water, and was used with the small curved probang. The expectorated membranes were exhibited.—*From Record of Bost. Soc. of Med. Improvement. American Jour. Med. Sciences.*

